# Dichotomous roles of IL-36 and IL-38 in cardiovascular disease

**DOI:** 10.3389/fimmu.2025.1642116

**Published:** 2025-07-08

**Authors:** Haifeng Zhang, Wenhui Huang, Shisan Bao

**Affiliations:** Department of Cardiology, The First People’s Hospital of Baiyin, Baiyin, Gansu, China

**Keywords:** IL-36, IL-38, cardiovascular disease, biomarker, anthogenesis

## Abstract

Members of the interleukin-1 (IL-1) superfamily play crucial roles in orchestrating inflammation and immune responses. Among them, IL-36 and IL-38 have emerged as cytokines with contrasting roles in cardiovascular disease (CVD). IL-36 typically promotes inflammation, contributing to endothelial dysfunction, atherogenesis, and myocardial injury. In contrast, IL-38 exerts predominantly anti-inflammatory effects, modulating immune responses and promoting tissue repair. This mini-review provides a critical synthesis of current findings on IL-36 and IL-38 in the context of atherosclerosis, myocardial ischaemia–reperfusion (I/R) injury, and post-percutaneous coronary intervention (PCI) outcomes. We discuss their molecular mechanisms, potential as biomarkers, and therapeutic implications, while identifying key gaps in knowledge that merit further investigation.

## Introduction

Atherosclerosis, the underlying cause of most cardiovascular diseases (CVD), is an autoimmune-mediated chronic inflammatory condition characterized by the accumulation of lipids, immune cells, and fibrous elements within the arterial wall (1). Endothelial dysfunction facilitates the infiltration of low-density lipoprotein (LDL) particles (2), which become oxidized and subsequently promote the recruitment of circulating monocytes and T cells. These activated immune cells, together with stimulated vascular smooth muscle cells (3), drive plaque formation and progression. Over time, unstable plaques may rupture in response to further stimuli (4), leading to thrombosis and clinical events such as myocardial infarction or stroke.

The IL-1 cytokine family includes a diverse set of pro-inflammatory and anti-inflammatory mediators such as IL-1α, IL-1β, IL-18, IL-33, IL-36α/β/γ, IL-37, and IL-38. These cytokines act through IL-1 receptors to activate downstream signaling cascades, notably the nuclear factor-kappa B (NF-κB) and mitogen-activated protein kinase (MAPK) pathways (5).

The IL-36 subfamily—comprising IL-36α, IL-36β, and IL-36γ—binds a heterodimeric receptor complex formed by IL-36R and the IL-1 receptor accessory protein (IL-1RAcP), promoting inflammatory signaling. Their endogenous antagonist, IL-36Ra, competes for receptor binding but fails to recruit IL-1RAcP, thereby blocking downstream effects (6). Produced predominantly by epithelial cells, IL-36 cytokines act on immune and stromal cells and require N-terminal proteolytic processing to become fully active. Their roles in chronic inflammatory diseases (7, 8) and cancer are increasingly recognized (9, 10).

In contrast, IL-38 is a more recently identified, anti-inflammatory member of the IL-1 family that suppresses both innate and adaptive immune responses (11, 12). IL-38 shares structural similarity with IL-36Ra and IL-1Ra and can antagonize their respective receptors. Its expression is upregulated in autoimmune diseases such as rheumatoid arthritis (13) and inflammatory bowel disease (14), but is suppressed in certain cancers, such as colorectal carcinoma (15) or prostate cancer (16). In CVD, IL-1 family cytokines influence endothelial dysfunction, myocardial injury, and vascular remodeling (17).

While IL-32, IL-34, and IL-37 have received attention in the CVD—showing abnormal expression in atherosclerotic plaques, with IL-32 and IL-34 exhibiting pro-atherogenic properties linked to unstable plaque phenotypes, and IL-37 playing an anti-atherogenic role by contributing to plaque stability (18) —the specific functions of IL-36 and IL-38 remain poorly defined.

Notably, despite their shared lineage, IL-36 and IL-38 often exert opposing effects-pro-inflammatory and anti-inflammatory, respectively—raising important questions about their roles in cardiovascular pathology.

Additionally, IL-6 is a multifunctional cytokine that plays a pivotal role in both acute-phase responses and the regulation of chronic inflammation (19). In atherogenesis, IL-6 is secreted by endothelial cells, macrophages, and vascular smooth muscle cells in response to stimuli such as oxidized LDL and other pro-inflammatory signals (20). Elevated IL-6 levels are implicated in endothelial dysfunction, enhanced monocyte recruitment, and the stimulation of C-reactive protein synthesis—an established biomarker of cardiovascular risk. Through its pro-inflammatory and pro-atherogenic effects, IL-6 contributes to the progression and destabilization of atherosclerotic plaques, ultimately increasing the likelihood of plaque rupture, thrombosis, and subsequent cardiovascular events (20, 21).

This mini-review focuses on IL-36 and IL-38 in the context of cardiovascular disease. We summarize current knowledge on their divergent roles in vascular inflammation, atherosclerosis, and ischemic injury, highlighting their emerging promise as diagnostic markers and potential therapeutic targets.

## Pro-inflammatory roles of IL-36 in cardiovascular pathology

The IL-36 cytokines—IL-36α, IL-36β, and IL-36γ—are pro-inflammatory ligands that bind to the IL-36 receptor (IL-36R), activating downstream NF-κB and MAPK pathways (5). These cytokines are produced by epithelial cells, monocytes, dendritic cells, and endothelial cells in response to inflammatory stimuli (22).

Clinical studies have associated elevated IL-36 levels with CVD. Kazemian et al. reported significantly increased circulating IL-36 levels in CVD patients compared with healthy controls (23), with positive correlations observed with pro-inflammatory mediators (TNF, IL-6, IL-32) and lipid markers (total cholesterol, oxLDL), alongside a negative correlation with antioxidant capacity, as measured by the ferric reducing ability of plasma (FRAP) (24). These findings suggest that IL-36 may contribute to atherosclerosis development by promoting systemic inflammation, e.g. IL-6, and oxidative stress-mediated vascular injury. However, further studies are required to determine which IL-36 isoforms are specifically involved in the initiation and progression of atherosclerosis in human plaques.

However, further studies are needed to clarify which IL-36 isoforms are specifically involved in the initiation and progression of atherosclerosis in human plaques, as has been demonstrated for other cytokines (4, 25). Most existing studies have focused on IL-36γ, with limited data available for IL-36α and IL-36β. Future research should therefore investigate the differential expression of IL-36α, IL-36β, and IL-36γ at the protein and/or mRNA levels using immunohistochemistry and qRT-PCR, in order to characterize their expression patterns and elucidate the specific roles of each isoform.

Furthermore, the role of IL-36 has also been investigated in ApoE^-/-^ mice—a well-established model of atherosclerosis—in a dose- and time-controlled manner (26). IL-36γ expression was found to be upregulated in atherosclerotic lesions, particularly in high-fat diet-fed mice, at both the mRNA and protein levels (26). Expression was notably higher in advanced atheromatous plaques compared to early-stage lesions, consistent with increased circulating IL-36γ levels in the same model (26).

Macrophages, which play a central role in the pathogenesis of atherosclerosis in both humans and animal models (27), were identified as the predominant infiltrating leukocyte population in these plaques. Administration of exogenous IL-36γ to high-fat diet-fed ApoE^-/-^ mice resulted in significantly larger atheroma formation (26), accompanied by increased macrophage infiltration, with no corresponding rise in CD3^+^ T cells. This observation aligns with previous reports that macrophage infiltration predominates during early atherogenesis, whereas CD3^+^ T cell accumulation typically occurs in later stages (4, 27).

RNA-seq analysis revealed 511 differentially expressed genes (DEGs), including 169 upregulated and 342 downregulated genes. Several of these, including *Ccl12*, *Ccl5*, *Ldlr*, and *Cxcl3*, were implicated in the PI3K-Akt and NF-κB signaling pathways (26). Gene Ontology (GO) analysis of macrophages isolated from IL-36γ-treated plaques showed enrichment in pathways related to inflammatory responses, cell adhesion, and LDL particle binding, suggesting that IL-36γ modulates macrophage transcriptomic profiles and promotes the expression of pro-inflammatory mediators.

Collectively, these findings indicate that IL-36 may contribute to atherogenesis by initially promoting macrophage recruitment and activation (as precursors of foam cells), followed by the autocrine and paracrine release of inflammatory mediators.

Moreover, the IL-1 receptor accessory protein (IL-1RAcP), a co-receptor for IL-36R, is highly expressed in endothelial cells and infiltrating leukocytes within human atherosclerotic plaques, but not in smooth muscle cells (28). This distribution pattern supports a pro-atherogenic role for IL-36 in plaque development, potentially through the upregulation of endothelial adhesion molecules such as ICAM and ECAM, thereby facilitating leukocyte recruitment and promoting plaque progression. *In vitro*, treatment of human umbilical vein endothelial cells (HUVECs) with anti-IL-1RAcP reduced the expression of leukemia inhibitory factor (LIF), chemokine C-C motif ligand 4 (CCL4), and monocyte chemotactic protein 3 (MCP-3).

In more detail, leukemia inhibitory factor (LIF), a multifunctional cytokine of the IL-6 superfamily, plays an important role in host immunity and contributes to atheroma formation (29), consistent with findings that inhibition of LIF reduces atherosclerosis (30). C-C motif chemokine ligand 4 (CCL4), also known as macrophage inflammatory protein-1β (MIP-1β), promotes the migration and activation of natural killer (NK) cells (31) and contributes to plaque instability (32). Furthermore, monocyte chemotactic protein 3 (MCP-3) has also been shown to promote plaque instability (33). Therefore, downregulation of these pro-inflammatory mediators may help mitigate the development and progression of atherosclerosis. These findings provide further evidence that IL-36 signaling contributes to the development of atherosclerosis by regulating these molecules (28).

Inhibition of IL-36R suppresses activation of the NOD-like receptor pyrin domain-containing 3 (NLRP3) inflammasome, reduces plaque size, and improves plaque stability in murine models (28). The NLRP3 inflammasome is a crucial component of the innate immune system, acting as a sensor of cellular damage and infection (34). Upon activation, it triggers a cascade that leads to the release of pro-inflammatory cytokines such as IL-1β and IL-18, ultimately promoting inflammation. While this process is essential for pathogen clearance and tissue repair, its dysregulation contributes to various inflammatory diseases (34). This supports earlier evidence that targeting the IL-36 receptor reduces atherosclerosis by downregulating NLRP3 inflammasome activation (35). In aged mice, IL-36 neutralization alleviated coronary microvascular dysfunction and reduced infarct size following myocardial ischaemia-reperfusion injury, further highlighting IL-36R as a promising therapeutic target for atheroma management. However, further validation in human studies—preferably large, multicenter trials—is necessary.

Estrogen and other female sex hormones are known to confer protection against the development of atherosclerosis (36, 37). Supporting this, El-Awaisi et al. reported significantly higher expression of IL-36α, IL-36β, and IL-36γ in female human hearts compared to male hearts (38), suggesting a possible atheroprotective role for IL-36 modulated by female hormones. In a myocardial ischaemia–reperfusion injury model, female mice showed increased neutrophil recruitment, whereas male mice exhibited a greater thrombotic burden (38). Male mice also had lower capillary density and a reduced capacity to restore perfusion, while females exhibited improved perfusion recovery but developed larger infarcts. Notably, treatment with IL-36 receptor antagonist (IL-36Ra) reduced inflammation, improved perfusion, and decreased infarct size in both sexes, despite increased platelet accumulation in male hearts. These benefits were attributed to IL-36Ra’s ability to reduce endothelial oxidative stress and suppress VCAM-1 expression. The timing of IL-36Ra administration during the ischemic phase was found to be critical in achieving vasculoprotective effects (38).

Most of the studies discussed above rely on single animal models or *in vitro* systems, and their findings are yet to be translated into clinical applications. Several key uncertainties remain, including the precise cellular sources of IL-36 isoforms in vascular tissues, their specific roles in acute versus chronic inflammation, and their interactions with other cytokines *in vivo*, particularly during the development of atheroma—all of which merit further investigation.

While experimental evidence supports a protective role for female sex hormones, clinical findings remain inconclusive. Large randomized trials have generally failed to demonstrate cardiovascular benefits of estrogen therapy, and guidelines in 2001 do not recommend hormone replacement therapy (HRT) for the prevention of CVD (39). However, more recent data from a substantial body of randomized clinical trials—alongside observational studies, animal models, and basic research—suggest that the cardiovascular effects of HRT may depend on the timing of initiation relative to menopause (40). The ELITE trial provided direct evidence supporting this “timing hypothesis,” while the DOPS trial demonstrated that initiating HRT around the time of menopause (in women with a mean age of 50 years and 7 months postmenopausal) conferred cardiovascular benefits with minimal associated risk when used long-term. These findings align with earlier observational studies.

Discrepancies between experimental and clinical findings—such as differences in short-term versus long-term effects, and single-center versus multicenter study designs—may reflect variability in genetic background, environmental exposures, and lifestyle factors. Therefore, further investigation is warranted to elucidate the precise role of sex hormones in the development and progression of atherosclerosis.

Moreover, most of the studies discussed above rely on single animal models or *in vitro* systems, and their findings have yet to be translated into clinical applications. Key uncertainties remain, including the specific cellular sources of IL-36 isoforms within vascular tissues, their differential roles in acute versus chronic inflammation, and the *in vivo* interactions with other cytokines during atheroma formation. These aspects warrant further detailed investigation.

## The role of interleukin-38 in cardiovascular diseases

In contrast to the pro-inflammatory IL-36 subfamily, IL-38 functions as an anti-inflammatory cytokine and appears to exert counter-regulatory effects within the IL-1 family. Inflammation is a key driver of CVD, with atherosclerosis serving as the underlying cause of many conditions, including myocardial infarction and aortic valve calcification. IL-38, a relatively understudied member of the IL-1 family, exhibits immunomodulatory properties by dampening inflammatory signaling cascades. This section summarizes emerging evidence supporting the protective role of IL-38 in cardiovascular health and disease.

### IL-38 and atherosclerosis

Atherosclerosis is a chronic inflammatory disease characterized by lipid accumulation, endothelial dysfunction, and immune cell infiltration. IL-38 has been implicated in mitigating atherosclerosis by attenuating inflammation-related signaling pathways, including MAPK and NF-κB (41). These anti-atherogenic effects may reduce the risk of cardiovascular events, highlighting IL-38’s potential as both a biomarker and therapeutic target.

IL-38 has been detected in human atheromatous plaques, suggesting a role in disease modulation. It acts in part by antagonizing the IL-36 receptor and inhibiting NF-κB and AP-1 signaling pathways. Additionally, in experimental models, transfer of the IL-38 gene into bone marrow-derived mesenchymal stem cells (MSCs) in ApoE-/- mice *via* an adenoviral vector led to reduced plaque burden and systemic inflammation, without the adverse effects commonly associated with statin therapy (41), suggesting strong potential for clinical application in precision medicine.

Structurally, IL-38 shares homology with IL-1Ra and IL-36Ra, and is primarily expressed by macrophages, B cells, and dendritic cells. It signals through IL-36R and IL-1RAPL1 (11, 42). In atherosclerotic models, exogenous IL-38 reduces expression of endothelial adhesion molecules (VCAM-1, ICAM-1) and pro-inflammatory cytokines, limiting monocyte/macrophage infiltration. These findings suggest a multifaceted anti-inflammatory mechanism relevant to atherogenesis.

### IL-38 in myocardial ischaemia–reperfusion injury

Myocardial ischaemia–reperfusion (I/R) injury exacerbates cardiac damage following ischemic events. IL-38 has been shown to attenuate I/R injury by suppressing macrophage-driven inflammation (43). Specifically, it promotes M2 polarization of macrophages, inhibits activation of NLRP3 inflammasome, and enhances secretion of anti-inflammatory cytokines such as IL-10 and TGF-β. Polarization of M0 macrophages into M1 (pro-inflammatory) or M2 (anti-inflammatory) phenotypes plays a critical role in host immunity within the local environment (44), which in turn influences the progression and outcome of local atheroma formation (45, 46). IL-38 also interacts with IL-1RAPL1 to activate the JNK/AP-1 pathway, thereby increasing IL-6 production and promoting dendritic cell-induced regulatory T cell (Treg) responses (47)—collectively contributing to improved ventricular remodeling post-infarction (43).

### IL-38 in aortic valve calcification

Calcific aortic valve disease (CAVD) is the most prevalent valvular disorder among the elderly, particularly in patients with rheumatoid arthritis or age-related degeneration (48). It frequently progresses to aortic stenosis, which can result in heart failure or sudden cardiac death (49). Despite extensive research, no pharmacological treatment has been shown to halt or reverse CAVD progression—leaving valve replacement as the only effective intervention.

Chronic inflammation plays a central role in CAVD pathogenesis. Valve interstitial cells (VICs), which reside in all layers of the aortic valve, can undergo inflammation-driven osteogenic differentiation, leading to calcium deposition. Inflammatory hallmarks of CAVD include immune cell infiltration and extracellular matrix remodeling (49).

Recent evidence suggests a protective role for IL-38 in CAVD. Its expression is significantly reduced in calcified human aortic valves compared to non-calcified controls (50). Exogenous IL-38 treatment in VICs downregulates ICAM-1, VCAM-1, and RUNX2 expression, and reduces calcium deposition, likely *via* suppression of NLRP3 inflammasome activation and caspase-1 activity. *In vivo* models further support these findings, with IL-38 deficiency accelerating valve calcification. These data indicate that IL-38 inhibits both inflammatory and osteogenic responses and may serve as a novel therapeutic candidate in CAVD.

Following an extensive literature search, no reports were found regarding the involvement of IL-36 in CAVD. However, given the opposing role of IL-36 relative to IL-38, it is speculated that IL-36 may contribute to the progression of CAVD due to its pro-inflammatory properties. Specifically, IL-36 may upregulate adhesion molecules such as ICAM-1 and VCAM-1 on the endothelial surface, thereby promoting the recruitment of leukocytes to affected areas, including the aortic valves. Nevertheless, this hypothesis requires further validation through studies using clinical samples and animal models.

### IL-38 and major adverse cardiovascular events post-PCI

Percutaneous coronary intervention (PCI) is widely used to restore coronary perfusion in patients with coronary heart disease (CHD), yet major adverse cardiovascular events (MACE) remain a significant post-procedural risk. Retrospective studies have identified low serum IL-38 levels as an independent predictor of MACE after PCI (51). In patients with ST-elevation myocardial infarction (STEMI), those with low plasma IL-38 levels had a significantly higher incidence of MACE (23.7%) compared to those with high IL-38 levels (7.8%) (52).

Multivariate analysis revealed that post-PCI MACE risk correlated with smoking, HbA1c, HDL-C, and IL-38 levels, but not with age, hypertension, or baseline lipid biochemistry. Receiver operating characteristic (ROC) analyses confirmed the specificity and sensitivity of IL-38 as a prognostic biomarker across subgroups stratified by smoking status, serum HbA1c, and hs-CRP. These findings highlight the potential of IL-38 as a non-invasive biomarker for risk stratification following PCI.

However, there is currently no evidence linking IL-38 to sex hormone regulation or sex-specific cardiovascular effects, as has been discussed for IL-36 above, which warrants further investigation.

## Conclusion

IL-38 is a promising anti-inflammatory cytokine with protective effects across multiple cardiovascular conditions, including myocardial I/R injury, atherosclerosis, aortic valve calcification, and post-PCI outcomes. Its functions include promoting M2 macrophage polarization, inhibiting inflammatory signaling, and supporting tissue repair. However, important questions remain regarding receptor specificity, cell type–dependent effects, and crosstalk with metabolic and fibrotic pathways. Further mechanistic and translational research is warranted to clarify these roles and explore IL-38 as a therapeutic target in cardiovascular medicine.

## Limitations and future directions

While IL-36 and IL-38 show therapeutic promise in atherosclerosis, myocardial ischaemia-reperfusion injury, and post-PCI outcomes, most supporting evidence remains preclinical, derived largely from *in vitro* studies or single-model animal experiments, limiting direct clinical translation. These cytokines exemplify the contrasting roles of immune mediators in cardiovascular inflammation: IL-36 generally drives pro-inflammatory responses and disease progression, whereas IL-38 mitigates inflammation and promotes tissue repair. Advancing their clinical application will require deeper mechanistic insights, better patient stratification, and innovative therapeutic strategies.

Future research should focus on identifying upstream regulators of IL-36 and IL-38 in cardiovascular tissues, clarifying receptor- and cell-type-specific signaling, and evaluating their clinical relevance as biomarkers and therapeutic targets through large-scale longitudinal studies. Concurrently, advanced delivery methods—such as bioengineered MSCs or targeted mimetics—may enhance the safety and precision of cytokine-based therapies. Targeting IL-36 and IL-38 holds promise for personalized cardiovascular immunotherapy tailored to individual inflammatory profiles and disease stages.
